# Evolution of Developmental Programs for the Midline Structures in Chordates: Insights From Gene Regulation in the Floor Plate and Hypochord Homologues of *Ciona* Embryos

**DOI:** 10.3389/fcell.2021.704367

**Published:** 2021-06-21

**Authors:** Kouhei Oonuma, Maho Yamamoto, Naho Moritsugu, Nanako Okawa, Megumi Mukai, Miku Sotani, Shuto Tsunemi, Haruka Sugimoto, Eri Nakagome, Yuichi Hasegawa, Kotaro Shimai, Takeo Horie, Takehiro G. Kusakabe

**Affiliations:** ^1^Department of Biology, Faculty of Science and Engineering, Konan University, Kobe, Japan; ^2^Institute for Integrative Neurobiology, Graduate School of Natural Science, Konan University, Kobe, Japan; ^3^Shimoda Marine Research Center, University of Tsukuba, Shimoda, Japan

**Keywords:** ascidian, Sonic hedgehog, floor plate, endodermal strand, *Ciona intestinalis* sp. A, notochord, FoxA transcription factors, hypochord

## Abstract

In vertebrate embryos, dorsal midline tissues, including the notochord, the prechordal plate, and the floor plate, play important roles in patterning of the central nervous system, somites, and endodermal tissues by producing extracellular signaling molecules, such as Sonic hedgehog (Shh). In *Ciona*, *hedgehog.b*, one of the two *hedgehog* genes, is expressed in the floor plate of the embryonic neural tube, while none of the *hedgehog* genes are expressed in the notochord. We have identified a *cis*-regulatory region of *hedgehog.b* that was sufficient to drive a reporter gene expression in the floor plate. The *hedgehog.b cis*-regulatory region also drove ectopic expression of the reporter gene in the endodermal strand, suggesting that the floor plate and the endodermal strand share a part of their gene regulatory programs. The endodermal strand occupies the same topographic position of the embryo as does the vertebrate hypochord, which consists of a row of single cells lined up immediately ventral to the notochord. The hypochord shares expression of several genes with the floor plate, including *Shh* and *FoxA*, and play a role in dorsal aorta development. Whole-embryo single-cell transcriptome analysis identified a number of genes specifically expressed in both the floor plate and the endodermal strand in *Ciona* tailbud embryos. A *Ciona* FoxA ortholog FoxA.a is shown to be a candidate transcriptional activator for the midline gene battery. The present findings suggest an ancient evolutionary origin of a common developmental program for the midline structures in Olfactores.

## Introduction

The embryonic midline tissues, notochord, and floor plate are signaling centers that pattern vertebrate embryos (Placzek and Briscoe, 2005; Stemple, 2005). The notochord acts as an axial supportive structure and induces the floor plate in the neural tube and patterns somitic mesoderm via Sonic hedgehog (Shh) secretion. The floor plate then patterns the neural tube along the dorso-ventral axis also using the Shh signal. Thus these midline structures are central elements for construction of the vertebrate body plan.

In anamniote embryos, an endodermal rod-shaped structure, hypochord, transiently appears ventral to the notochord (Franz, 1898; Reinhardt, 1904; Gibson, 1910). Development of the hypochord also depends on signals from the notochord (Cleaver and Krieg, 1998). The hypochord was once thought to be simply a supportive structure (Stöhr, 1895; Corbo et al., 1997a) but several lines of evidence suggest that it plays a role in the positioning of the dorsal aorta (Cleaver et al., 1997; Löfberg and Collazo, 1997; Cleaver and Krieg, 1998; Eriksson and Löfberg, 2000; Hogan and Bautch, 2004), and for determination of left–right axis asymmetry (Danos and Yost, 1996; Lohr et al., 1997). Thus, transient midline tissues originating from different germ layers, the floor plate (ectoderm), the notochord (mesoderm), and the hypochord (endoderm), pattern the embryonic structure in vertebrates.

The hypochord shares expression of several genes with the floor plate and the notochord, including *Shh* and *FoxA* (Yan et al., 1995; Appel et al., 1999; Dal-Pra et al., 2011; Peyrot et al., 2011). Although their originating germ layers are different, progenitor cells of these midline tissues locate close to one another in the dorsal marginal zone, such as the Spemann organizer in amphibians and the embryonic shield in zebrafish (Shih and Fraser, 1995; Melby et al., 1996; Latimer et al., 2002; Latimer and Appel, 2006; Dal-Pra et al., 2011; Peyrot et al., 2011). These commonalities suggest a tight developmental and evolutionary connection among these midline structures. The notochord is the organ that define the phylum (or superphylum) Chordata, including vertebrates, tunicates, and cephalochordates (Kowalevsky, 1866, 1867; Yasuo and Satoh, 1993; Corbo et al., 1997a, b; Satoh et al., 2014). The ventral midline of the neural tube (nerve cord) in tunicate *Ciona* embryos expresses homologues of *Shh* (*hedgehog.b*) and *FoxA* (*FoxA.a*), and has been identified as the floor plate homologue (Corbo et al., 1997a; Takatori et al., 2002; Shi et al., 2009). By contrast, the presence of a hypochord homologue remains obscure in invertebrate chordates, although it has been suggested to be homologous with the epibranchial groove of amphioxus (Klaatsch, 1898) and a similarity between the hypochord and the endodermal strand of *Ciona* embryos has been pointed out (Corbo et al., 1997a).

Here we provide new evidence that the endodermal strand shares the gene regulatory mechanism with the floor plate in *Ciona* embryos. Functional analysis of the *cis*-regulatory region of the floor plate-specific *hedgehog.b* gene revealed its latent ability to drive transcription in the endodermal strand. Whole-embryo single-cell transcriptome analysis identified a number of genes specifically expressed in both the floor plate and the endodermal strand in *Ciona* tailbud embryos. These genes and their transcriptional regulation suggest an ancient evolutionary origin of a common developmental program for the midline structures in Olfactores. Our findings also support homology between the vertebrate hypochord and the tunicate endodermal strand.

## Results and Discussion

### Transcriptional Activation by *Cis*-Regulatory Regions of *Ciona hedgehog.b* in the Floor Plate and Hypochord Homologues

**FIGURE 1:**
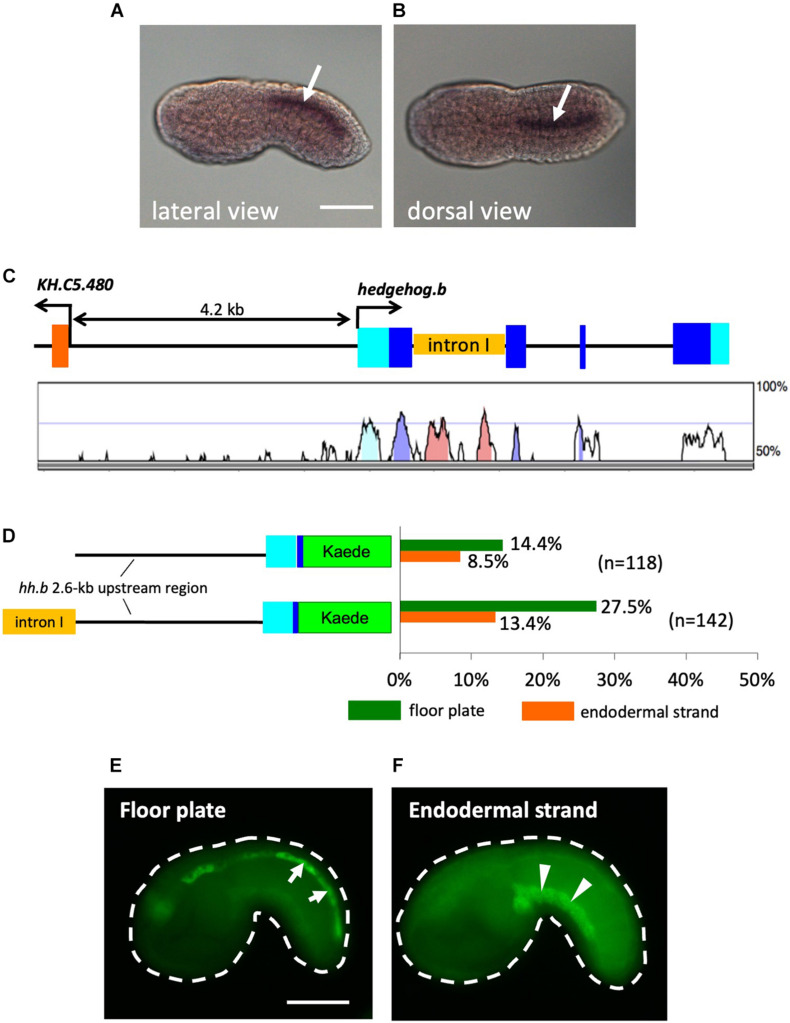
Activity of *cis*-regulatory regions of *Ciona hedgehog.b* in the floor plate and the endodermal strand of tail bud embryos. **(A,B)** Expression pattern of *hedgehog.b* at the tailbud stage visualized by whole-mount *in situ* hybridization. Anterior is to the left. *Arrows* indicate the floor plate (ventral nerve cord). **(A)** Lateral view. **(B)** Dorsal view. **(C)** Genomic organization of the *hedgehog.b* gene. The exons are indicated by boxes in cyan (untranslated regions) and blue (coding regions). The untranscribed regions and introns are indicated by lines. A peaks-and-valleys graph below the genomic organization diagram is a VISTA profile (Frazer et al., 2004) showing the percent conservation of the nucleotide sequence of each region between *C. intestinalis* type A and *Ciona savignyi*. **(D)** The structure of the Kaede reporter constructs and the reporter expression patterns observed. The left diagrams show schematic structure of each construct. Cyan and blue boxes indicate the 5′ untranslated region and a partial coding region, respectively, of *hedgehog.b*. The orange box indicates the first intron sequence of *hedgehog.b*. Bars in the right graph show the percentage of larvae with Kaede expression in a tissue out of all electroporated embryos scored for each construct. Different colors of bars represent expression in different tissues: green, floor plate; orange, endodermal strand. Numbers in parentheses indicate the number of larvae scored for each construct. **(E,F)** Examples of mid tailbud embryos electroporated with the *hedgehog.b*(+int) > *kaede* construct showing Kaede expression in the floor plate (arrows in panel **E)** and the endodermal strand (arrowheads in panel **F)**. Scale bars, 50 μm.

*Ciona hedgehog.b* is expressed in the floor plate, but not in the notochord during embryogenesis (Takatori et al., 2002; Islam et al., 2010; Figures 1A,B). When the 2.6-kb upstream region of *hedgehog.b* connected with a Kaede reporter (*hedgehog.b* > *kaede*) was introduced into *Ciona* embryos, the expression of Kaede reporter was observed in the floor plate at the mid tailbud stage (Figure 1D). In addition to the expression in the floor plate, “ectopic” Kaede expression was observed in the endodermal strand of some embryos (Figure 1D). In contrast, no Kaede expression was observed in the notochord.

Because the reporter expression was only observed in a relatively small proportion of transfected embryos and the ectopic expression in the endodermal strand was observed (Figure 1D), we thought that additional *cis*-regulatory sequences might be present outside of the 2.6-kb upstream region. Comparative genomics between *Ciona intestinalis* type A and *Ciona savignyi* revealed that the first intron of *hedgehog.b* contains highly conserved non-coding regions, which could be candidates for such additional *cis*-regulatory sequences (Figure 1C). To test this possibility, we placed the first intron sequence upstream of the 2.6-kb genomic region in the *hedgehog.b* > *kaede* construct and examined Kaede reporter expression in embryos transfected with this DNA construct (Figure 1D). As expected, higher frequency of Kaede expression in the floor plate was observed (Figures 1D,E). However, the reporter expression in the endodermal strand also remained (Figures 1D,F). The endodermal strand is a caudal midline structure that lies immediately ventral to the notochord and its homology with the vertebrate hypochord has been proposed (Corbo et al., 1997a). Thus, the *cis*-regulatory regions of *Ciona hedgehog.b* can activate transcription in the floor plate and hypochord homologues. This observation further prompted us to test an idea that the floor plate and the endodermal strand share a developmental program including the transcriptional machinery.

### Single-Cell Transcriptomic Analysis Revealed a Gene Battery Shared Among the Midline Tissues

**FIGURE 2:**
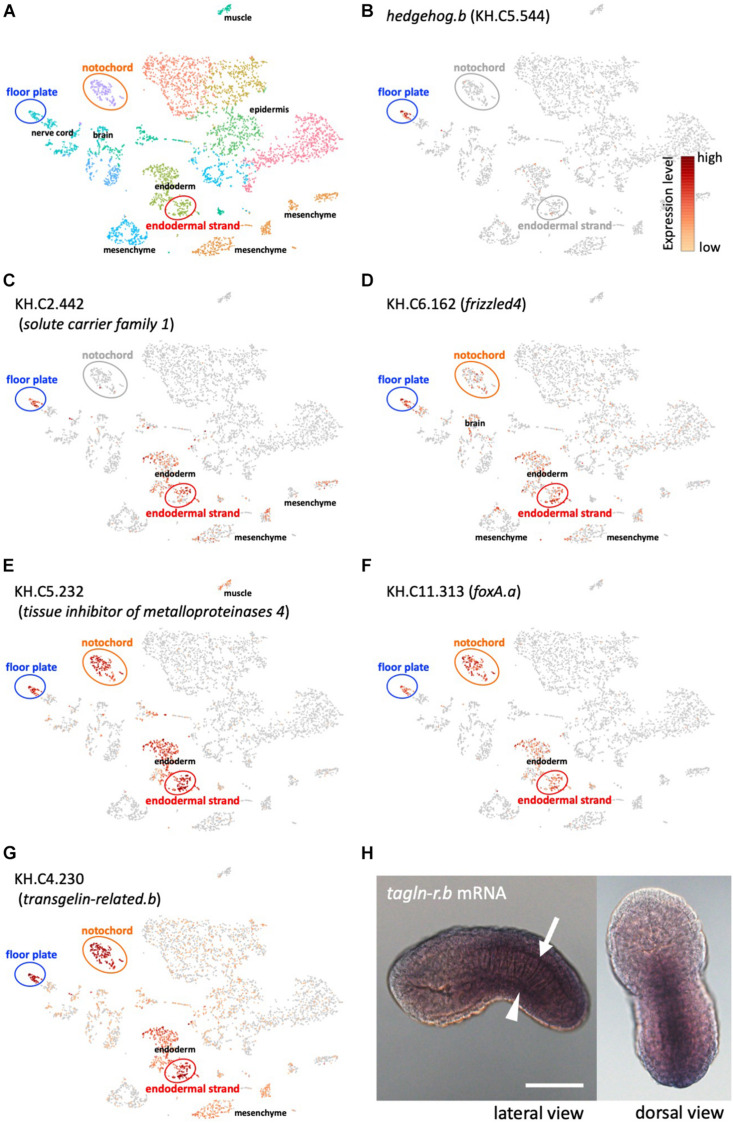
Whole-embryo single-cell RNA-seq analysis of midline tissue-specific genes. **(A)** A t-distributed stochastic neighbor embedding (t-SNE) projection map of mid-tailbud stage embryos obtained in a previous study (Horie T. et al., 2018). Each dot corresponds to the transcriptome of a single cell, and cells possessing similar transcriptome profiles map near each other. The major tissue types in tailbud-stage embryos were identified. Identification of tissue types is based on the expression of known marker genes as previously described (Horie T. et al., 2018). Clusters of cells corresponding to the floor plate, the notochord, and the endodermal strand are encircled. **(B)** The t-SNE projection map highlighting *hedgehog.b*-expressing cells (red dots) in the floor plate. **(C–G)** The t-SNE projection map showing the expression profiles of representative genes that are specifically expressed in both the floor plate and the endodermal strand (Table 1). Red and orange dots indicate cells expressing KH.C2.442 **(C)**, KH.C6.162 (*frizzled4*, **D)**, KH.C5.232 **(E)**, KH.C11.313 (*foxA.a*, **F)**, and KH.C4.230 (*tagln-r.b*, **G)**. **(H)** Expression pattern of *tagln-r.b* at the tailbud stage visualized by whole-mount *in situ* hybridization. The *arrow* and the *arrowhead* indicate the floor plate and the endodermal strand, respectively. Scale bar, 50 μm.

To further investigate the shared developmental program between the floor plate and the endodermal strand, we compared gene expression profiles between these tissues by whole-embryo single-cell transcriptomics at the mid tailbud stage (Table 1 and Figure 2; Horie T. et al., 2018; Horie R. et al., 2018; Cao et al., 2019). Whole-embryo single-cell transcriptome data clearly revealed that *hedgehog.b* is expressed in the floor plate but not expressed in any other tissues, including the notochord and the endodermal strand (Figure 2B). Among the top 20 genes highly expressed in the endodermal strand, 8 genes were shown to be significantly enriched (*p* < 0.05) in the floor plate (Table 1). Of these, five genes were highly enriched (*p* < 0.001) in the floor plate (Table 1 and Figure 2). These genes include *fz4* (gene model ID KH.C6.162) encoding a Frizzled4 receptor, *foxA.a* (KH.C11.313) encoding a FoxA transcription factor, KH.C2.442 encoding a solute carrier family 1 protein, KH.C5.232 encoding a tissue inhibitor of metalloproteinases 4, and KH.C4.230 encoding a calponin/transgelin family protein (*transgelin-related.b*). Interestingly, four of these genes (*fz4*, *foxA.a*, KH.C5.23, and KH.C4.230) are also expressed in the notochord (Figures 2D–G). The expression pattern of *foxA.a* is consistent with the previously reported whole-mount *in situ* hybridization (Corbo et al., 1997a). These genes may constitute a gene battery co-regulated in the midline tissues at the mid tailbud stage.

**TABLE 1:** Top 20 upregulated genes in the endodermal strand at the mid tailbud stage.

Gene Model ID	Endodermal strand		Floor plate		Similarity or predicted gene product
	log_2_ fold change	*p*-value	log_2_ fold change	*p*-value	
KH2012:KH.L41.54	6.44	3.63E-35	−1.61	1	Zinc transporter ZIP1
KH2012:KH.C4.693	5.54	1.39E-30	−2.18	1	SLIT and NTRK-like protein
KH2012:KH.C9.162	3.76	2.90E-15	−0.27	1	Regulator of G-protein signaling
KH2012:KH.C9.672	3.59	1.13E-14	0.22	1	Regulator of G-protein signaling
KH2012:KH.C1.520	3.28	1.79E-14	−0.02	1	Secreted frizzled-related protein (sFRP3/4-b)
KH2012:KH.C5.232	3.26	6.66E-13	3.41	4.04E-05	Tissue inhibitor of metalloproteinases 4
KH2012:KH.L46.15	2.79	2.65E-10	0.61	1	Uncharacterized protein
KH2012:KH.C6.162	2.88	2.69E-08	3.99	4.12E-07	Frizzled receptor (Fz4)
KH2012:KH.C4.230	2.76	4.52E-08	4.22	3.96E-09	Transgelin/Calponin/Neuronal protein 25/SM22a (tagln-r.b)
KH2012:KH.C2.378	2.81	4.75E-08	3.00	3.42E-03	brain-enriched hyaluronan-binding protein
KH2012:KH.C6.37	2.77	5.59E-08	−4.91	1	P-loop containing nucleoside triphosphate hydrolases
KH2012:KH.C9.174	3.22	7.21E-08	−3.65	1	Hypothetical protein
KH2012:KH.C3.203	2.58	9.24E-08	2.34	4.92E-02	Sulfotransferase
KH2012:KH.C2.442	2.83	2.73E-07	4.18	1.62E-07	Solute carrier family 1 (glial high affinity glutamate transporter)
KH2012:KH.C10.229	2.51	3.56E-07	−4.57	1	Phosphatidylcholine transfer protein
KH2012:KH.C2.245	2.63	7.06E-07	−0.53	1	UDP-GlcNAc:betaGal beta-1,3-N-acetylglucosaminyltransferase 2
KH2012:KH.C11.313	2.53	2.85E-06	3.55	2.97E-05	Fork head/HNF-3 homologue (FoxA-a)
KH2012:KH.C9.229	2.27	4.50E-06	0.98	1	Nck-associated protein 5
KH2012:KH.L4.17	2.09	6.46E-05	−1.17	1	Zinc finger protein (Sal-like protein 1)
KH2012:KH.C3.585	2.13	7.16E-05	2.49	2.49E-02	SCRaMblase (phospholipid scramblase) family member (scrm-1)

**FIGURE 3:**
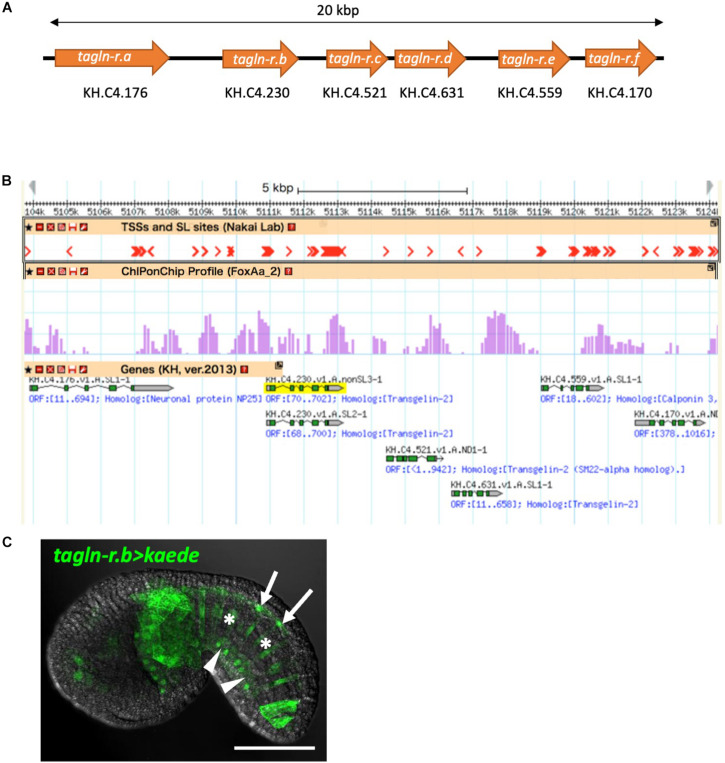
The calponin/transgelin family gene cluster in the *Ciona* genome. **(A)** Schematic diagram of the gene cluster. Six genes, *tagln-r.a*, *tagln-r.b*, *tagln-r.c*, *tagln-r.d*, *tagln-r.e*, and *tagln-r.f*, each encoding a calponin/transgelin family protein are clustered in a 20-kb genomic region. **(B)** The transcriptional landscape of the *tagln-r* loci. Transcription start sites (TSSs) and spliced leader (SL) *trans*-splicing sites (Yokomori et al., 2016) and FoxA.a binding sites determined by ChIP-on-chip analysis (Kubo et al., 2010) were mapped on the Ghost Genome Browser (Satou et al., 2005, 2008). **(C)** Localization of Kaede reporter expressed under the control of the upstream *cis*-regulatory region of *tagln-r.b*. *Arrows*, *arrowheads*, and *asterisks* indicate the floor plate, the endodermal strand, and the notochord, respectively. Scale bar, 50 μm.

For further analysis, we adopted KH.C4.230 as a model to investigate transcriptional regulation in the midline tissues because its expression level is relatively high and the enriched expression in the floor plate, the notochord, and the endodermal strand is strongly supported by the single-cell transcriptomic analysis (*p*-values, 3.96E-09, 1.49E-14, and 4.52E-08, respectively). KH.C4.230 encodes a protein belonging to the calponin/transgelin family. Calponins and transgelins are actin-associated proteins highly conserved from yeast to mammals (Prinjha et al., 1994; Goodman et al., 2003). We named KH.C4.230 as *transgelin-related.b* (*tagln-r.b*) based on the sequence similarity and genomic arrangement (Figure 3). Whole-mount *in situ* hybridization confirmed that *tagln-r.b* is expressed in the floor plate, the notochord, and the endodermal strand (Figure 2H).

In the genome of *C. intestinalis* type A, *tagln-r.b* is clustered in tandem with five other calponin/transgelin family genes within a 20-kb genomic region (Figure 3A). Whole-embryo single-cell transcriptome and high-throughput *in situ* hybridization data in the Ghost database (Satou et al., 2005) indicate that at least three of these *tagln-r* genes (*tagln-r.c*, *tagln-r.d*, and *tagln-r.e*) are also specifically expressed in the floor plate, the notochord, and the endodermal strand (Supplementary Figure 1; spatial expression patterns of *tagln-r.e* can be found at http://ghost.zool.kyoto-u.ac.jp/cgi-bin/photogetkh.cgi?inkey=CLSTR02020). Thus the clustered *tagln-r* genes are likely to be co-regulated as a member of the gene battery above mentioned.

### The Role of FoxA.a as a Common Transcriptional Activator for the Midline Gene Battery

**FIGURE 4:**
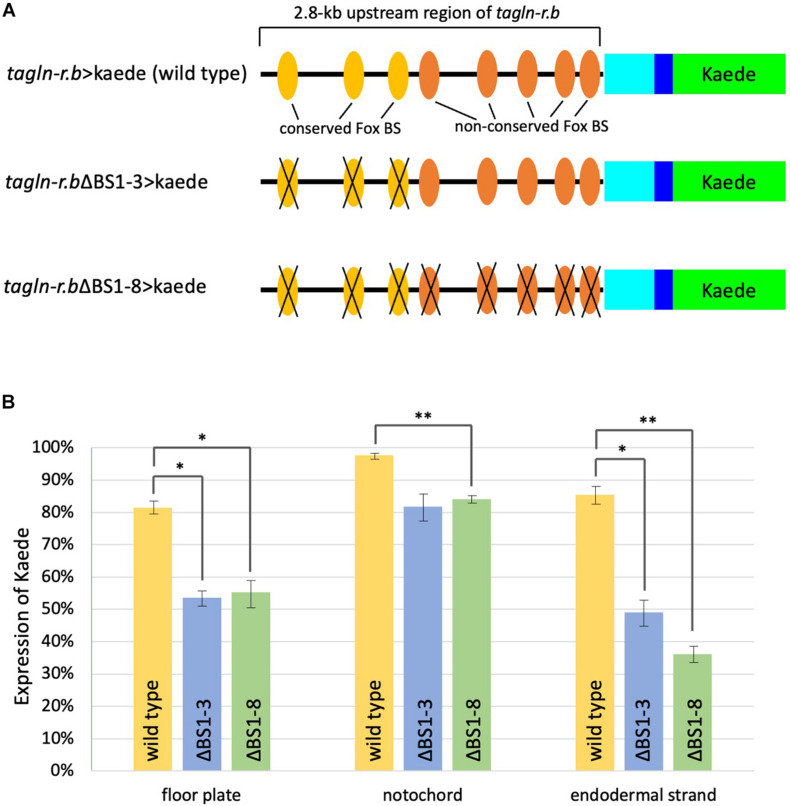
Functional analysis of putative Fox binding sites (BSs) in the *cis*-regulatory region of *tagln-r.b*. **(A)** Schematic diagram of the Kaede reporter constructs. Cyan and blue boxes indicate the 5′ untranslated region and a partial coding region, respectively, of *tagln-r.b*. Green boxes indicate the coding sequence of Kaede. Substitution mutations were introduced into putative Fox BSs. Colored ovals indicate the putative Fox BSs and black crosses indicate mutated BSs. **(B)** Expression of the Kaede reporter in the midline tissues of tailbud embryos. Localization of Kaede was detected by immunofluorescence staining in mid tailbud (12 hpf) embryos that developed from fertilized eggs electroporated with *tagln-r.b* > *kaede* fusion constructs. Vertical bars indicate the percentage of Kaede-positive embryos. Error bars represent SEM from three independent experiments. The total number of embryos scored for each construct was 172 for *tagln-r.b* > *kaede* (wild-type), 152 for *tagln-r.b*ΔBS1-3 > *kaede*, and 147 for *tagln-r.b*ΔBS1-8 > *kaede*. Statistical analysis was carried out using the standard Student *t*-test (^∗∗^*P* < 0.01, ^∗^*P* < 0.05).

Because the expression profile of *foxA.a* (Figure 2F) was very similar to that of *tagln-r.b* (Figure 2G), FoxA.a seemed to be a good candidate for a common transcriptional activator in the midline tissues. To test this possibility, we examined distribution of the FoxA.a binding sites in the upstream of the putative transcription start sites of each of the clustered *tagln-r* genes using a set of ChIP-on-chip data of FoxA.a (Kubo et al., 2010). As expected, FoxA.a binding sites are enriched in the 5′ flanking region of each *tagln-r* gene (Figure 3B). To analyze the transcriptional regulatory mechanism of *tagln-r.b*, its 2.8-kb upstream region was connected with the coding sequence of Kaede (Figure 4A) and introduced into *Ciona* embryos. The *tagln-r.b* > *kaede* DNA construct recapitulated the endogenous expression pattern of *tagln-r.b*; it was expressed in the floor plate, the notochord, and the endodermal strand (Figure 3C), suggesting that the 2.8-kb upstream region contains *cis*-regulatory sequences sufficient for transcription in the midline tissues.

The 2.8-kb upstream region of *tagln-r.b* contains eight putative Fox binding sites (Figure 4A). Among these sites, three distal sites [binding sites (BS) 1–3] are conserved between *C. intestinalis* type A and *Ciona savignyi*, whereas five proximal sites (BS4-8) are not conserved. To test functional importance of putative Fox BSs, three conserved sites (BS1-3) or all eight BSs (BS1-8) were mutated in the *tagln-r.b* > *kaede* construct (Figure 4A) and the reporter expression was examined in the mid tailbud embryos transfected with these DNA constructs (Figure 4B). When only the conserved sites were mutated (ΔBS1-3), the reporter expression was significantly reduced in the floor plate and the endodermal strand, whereas it was moderately reduced in the notochord. Additional mutations in the non-conserved BSs (BS4-8) did not further decrease the reporter expression in each tissue. These results suggest that a Fox transcription factor, presumably FoxA.a, serves as a transcriptional activator of *tagln-r.b* in the midline tissues via direct interaction with the upstream region. Our observation also suggests that a greater contribution of FoxA.a to transcriptional activation of *tagln-r.b* in the floor plate and the endodermal strand than in the notochord. Because disruption of all Fox BSs in the *cis*-regulatory region of *tagln-r.b* had only slightly reduced the reporter expression in the notochord (Figure 4), it is plausible that Brachyury is the main activator for *tagln-r.b* in the notochord.

Among 29 Fox transcription factors identified in *C. intestinalis* type A (Imai et al., 2004; Satou et al., 2005), FoxA.a is the most plausible candidate as the transcription factor that interacts with Fox BSs in the upstream region of *tagln-r.b* for three reasons. First, as mentioned above, the ChIP-on-chip data demonstrated FoxA.a binding to the upstream region of *tagln-r.b* (Kubo et al., 2010). Second, expression patterns of *foxA.a* and *tagln-r.b* are similar to each other. Third, none of the other Fox family genes show similar expression patterns (Imai et al., 2004). In a strict sense, however, the present analysis does not exclude the possibility that a Fox transcription factor other than FoxA.a is involved in the transcriptional activation of *tagln-r.b*. To further assess the role of FoxA.a in *tagln-r.b* expression in the midline tissues, functional manipulations of FoxA.a, such as overexpression of wild-type and a repressor form and tissue-specific knockdown, will be required in future studies.

Disruption of all Fox BSs in the *cis*-regulatory region of *tagln-r.b* did not completely abolished the reporter expression in the floor plate and the endodermal strand (Figure 4). This suggests that other transcription factors are involved in transactivation of *tagln-r.b*. Future identification of transcription factors that interacts with the *cis*-regulatory region of *tagln-r.b* will contribute to the elucidation of the gene regulatory networks for the development of the floor plate and the endodermal strand.

### Developmental Roles of the Endodermal Strand in *Ciona* Embryos

The hypochord, transient rod-like structure situated under the notochord, is first described in embryos of elasmobranchs (Leydig, 1852). Many morphological studies on this structure were reported in embryos of lampreys, fishes, and amphibians in the late 19th and early 20th centuries (Hatta, 1893; Franz, 1898; Klaatsch, 1898; Reinhardt, 1904; Gibson, 1910). Since then, however, the hypochord has been neglected by researchers for many years, and its function remains elusive. An inductive role in the formation of the dorsal aorta has been proposed (Cleaver et al., 1997; Löfberg and Collazo, 1997; Cleaver and Krieg, 1998; Eriksson and Löfberg, 2000). Although it is uncertain whether the hypochord has a structural counterpart in embryos of higher vertebrates, a similar inductive role of the dorsal endoderm in blood vessel patterning has been proposed in avian embryos (Hogan and Bautch, 2004).

**FIGURE 5:**
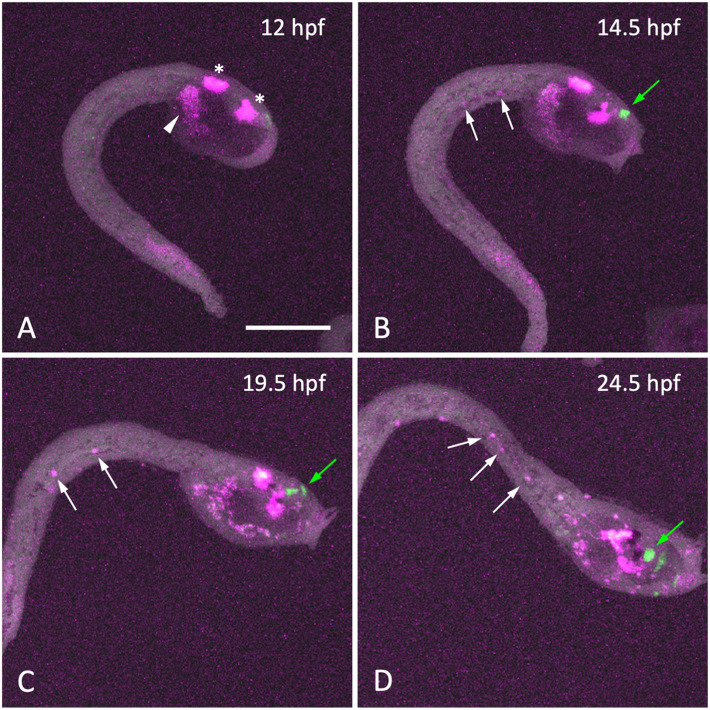
Migration of mesenchyme cells along the endodermal strand during larval development. **(A–D)** Time-lapse fluorescent images of a late-tailbud embryo expressing Kaede under the control of the *cis*-regulatory region of *Pax2/5/8.a* at the time indicated. Kaede fluorescence was photo-converted from green to red (shown in magenta) by 405-nm laser irradiation at 12 hpf **(A)**. At 12 hpf, photo-converted Kaede fluorescence was observed in the central nervous system (*asterisks*) and mesenchyme cells (*arrowhead*) in the trunk region, whereas no cells were labeled in the tail region. As development proceeded **(B–D)**, a few cells labeled with photoconverted-Kaede appeared in the tail region and posteriorly migrated along the endodermal strand (*white arrows*). Cells synthesized Kaede after photo-conversion were labeled with green fluorescence (*green arrows* in panels **B–D)**. Scale bar, 100 μm.

The only function of the endodermal strand known to date is its role as the precursor of the adult intestine (Hirano and Nishida, 2000; Nakazawa et al., 2013). The similarity between the hypochord and the endodermal strand prompted us to ask whether the *Ciona* endodermal strand has an inductive role similar to that of the vertebrate hypochord. In vertebrate embryos, the blood vessel precursor angioblasts migrate toward the hypochord or dorsal endoderm to form the dorsal aorta (Cleaver and Krieg, 1998; Eriksson and Löfberg, 2000; Hogan and Bautch, 2004). To test whether similar cell migration occurs in *Ciona* embryos, we labeled trunk mesenchyme cells with the photoconvertible fluorescent protein Kaede (Ando et al., 2002) and fluorescence emitted by Kaede was converted from green to red by irradiation with 405-nm violet light at 10 hpf. The *kaede* transgene was expressed using an upstream regulatory region of *Ciona pax2/5/8.a*, which could drive the reporter gene expression in trunk mesenchyme cells. These embryos were analyzed by time-laps imaging from late tailbud (12 hpf) to larval (24.5 hpf) stages (Figure 5). Some of the Kaede-labeled mesenchyme cells were shown to migrate into the tail along the endodermal strand (Figure 5 and Supplementary Video 1). The *Ciona* endodermal strand may exert an inductive cue for the migratory mesenchyme cells, suggesting a functional similarity between the vertebrate hypochord and the *Ciona* endodermal strand.

The top 10 predominantly expressed genes in the endodermal strand include genes encoding extracellular ligands and receptors, including SLIT and NTRK-like protein (KH.C4.693), secreted frizzled-related protein (KH.C1.520), and frizzled receptor (KH.C6.162) (Table 1). Expression of these genes suggests an active interaction between the endodermal strand and other tissues. In zebrafish, the hypochord expresses the *frzb*/*sfrp3* gene that encodes a secreted frizzled-related protein (Thisse et al., 2001; Tendeng and Houart, 2006), showing a further similarity between the endodermal strand and the hypochord. Functional analysis of these genes may give insights into the role of the endodermal strand in *Ciona* embryos.

### Conserved Developmental Programs for Midline Tissues in Olfactores

**FIGURE 6:**
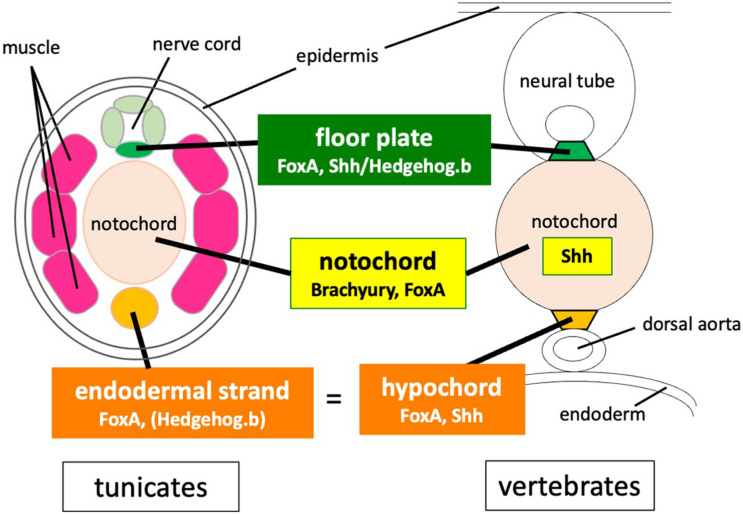
Comparison of developmental programs for midline tissues between tunicates and vertebrates. Co-expression of Brachyury and FoxA family transcription factors is required for notochord development both in vertebrates and tunicates. Both the notochord and the floor plate secrete Shh in vertebrate embryos, whereas *hedgehog* genes are not expressed in the notochord of tunicate embryos. *FoxA* and *Shh*/*hedgehog.b* are co-expressed in the floor plate of both vertebrates and tunicates. *FoxA* and *Shh* are also co-expressed in the hypochord precursor. The tunicate endodermal strand expresses FoxA.a and has a latent transactivation potential of *hedgehog.b*.

The present findings, along with a number of previous studies, illustrate common features and the difference of midline development between vertebrates and tunicates (Figure 6). The gene regulatory network for notochord development in ascidian embryos has been extensively studied (Imai et al., 2006; Hotta et al., 2008; Passamaneck et al., 2009; Kubo et al., 2010; José-Edwards et al., 2015; Reeves et al., 2021). *Brachyury* is a key specifier gene for the notochord formation. FoxA.a is an upstream activator of *Brachyury* (Imai et al., 2006; Hotta et al., 2008; Kubo et al., 2010), but it also directly activates notochord-specific genes (Passamaneck et al., 2009; José-Edwards et al., 2015; Reeves et al., 2021).

Co-expression of Brachyury and FoxA family transcription factors is required for notochord development both in vertebrates and tunicates (Herrmann and Kispert, 1994; Teillet et al., 1998; Friedman and Kaestner, 2006; Imai et al., 2006; Hotta et al., 2008; Passamaneck et al., 2009; José-Edwards et al., 2015). The notochord patterns the central nervous system, somitic mesoderm, and dorsal endoderm by secreting Shh in vertebrate embryos, whereas *hedgehog* genes are not expressed in the notochord of tunicate embryos (Takatori et al., 2002). FoxA and Shh/Hedgehog.b are co-expressed in the floor plate of both vertebrates and tunicates (Tessier-Lavigne et al., 1988; Placzek et al., 1990a, b; Takatori et al., 2002; Imai et al., 2009; Dal-Pra et al., 2011; Peyrot et al., 2011). FoxA and Shh are also co-expressed in the hypochord or its primordium (Ruiz i Altaba, 1998; Dal-Pra et al., 2011; Peyrot et al., 2011). The tunicate endodermal strand expresses *FoxA.a* (Corbo et al., 1997a) and has a latent transactivation potential of *hedgehog.b* as shown in this study.

In conclusion, the present study suggests that the floor plate and the hypochord homologue of *Ciona* embryos share a gene battery, which is regulated by a common transcription activator FoxA.a. The FoxA transcription factor seems to be a key regulator for midline development both in ascidians and vertebrates. The endodermal strand may have an inductive role for a novel population of migratory trunk cells, which further reveals a common feature shared between the endodermal strand and the hypochord. Altogether, the present findings suggest an ancient origin of a common developmental program for and common developmental roles of the midline structures in Olfactores.

## Materials and Methods

### *Ciona* Adults and Embryos

Mature adults of *C. intestinalis* type A (also called *Ciona robusta*) were provided by the Maizuru Fisheries Research Station of Kyoto University and by the Misaki Marine Biological Station of the University of Tokyo through the National Bio-Resource Project of the Ministry of Education, Culture, Sports, Science and Technology of Japan (MEXT), and were maintained in indoor tanks of artificial seawater (ASW) (Marine Art BR; Tomita Pharmaceutical, Tokushima, Japan) at 18°C. The adults were also collected from the pond on the Fukae campus of Kobe University, Kobe, Japan and from the fishing harbor in Murotsu, Hyogo, Japan. Eggs and sperm were obtained surgically from the gonoducts, and the eggs were fertilized *in vitro*. After insemination, the embryos were raised in ASW containing 50 μg/ml streptomycin sulfate (S6501; Sigma-Aldrich, St. Louis, MO, United States) at 18°C.

### Whole-Mount *in situ* Hybridization

The cDNA clones for *hedgehog.b* (Gene Collection ID R1CiGC41g11) and *tagln-r.b* (Gene Collection ID R1CiGC29n19) were obtained from the *Ciona* Gene Collection release 1 (Satou et al., 2002) and used as the templates to synthesize probes. To linearize the plasmid DNA for probe synthesis, cDNA clones were digested with *Xba*I (for *hedgehog.b*) or *Eco*RI (for *tagln-r.b*). Antisense RNA probes were synthesized with T7 RNA polymerase by using a DIG RNA Labeling Kit (Sigma-Aldrich, St. Louis, MI, United States). *Ciona intestinalis* type A embryos were fixed at the early tailbud stage in 4% paraformaldehyde in 0.1 M MOPS (pH 7.5) and 0.5 M NaCl at 4°C for 16 h, prior to storage in 80% ethanol at −30°C. Whole-mount *in situ* hybridization was carried out as described (Oonuma and Kusakabe, 2019).

### Preparation of Reporter Constructs and Electroporation

To make the *hedgehog.b > kaede* plasmid, the 2.6-kb upstream region of *Ciona hedgehog.b* (Gene Model ID KH.C5.544) was amplified from the genomic DNA of *C. intestinalis* type A by PCR using a pair of nucleotide primers (5′-ATCTGCAGGGTTTGATGCACAGCAAC-3′ and 5′-ATGGAT CCCCTGACCCGCATGATATGAC-3′), digested with *Pst*I and *Bam*HI, and then inserted into the *Pst*I/*Bam*HI sites of the pSP-Kaede vector (Hozumi et al., 2010). To make the *hedgehog.b*(+int) > *kaede* construct, the first intron sequence of *hedgehog.b* was amplified from the genomic DNA using a pair of nucleotide primers (5′-TTCTCGAGGCAGCAGTATGTGCCAC-3′ and 5′-CCCTGCA GCCATCCCAAGCTTCGATAAC-3′), digested with *Xho*I and *Pst*I, and then inserted into the *Xho*I/*Pst*I sites of the *hedgehog.b > kaede* plasmid. The *tagln-r.b* > *kaede* plasmid was made by inserting the 2.8-kb upstream region of *Ciona tagln-r.b* (Gene Model ID KH.C4.230) into the pSP-Kaede plasmid using an In-Fusion HD Cloning kit (Takara Bio, Japan). The 2.8-kb upstream region of *tagln-r.b* was amplified from the genomic DNA by PCR using a pair of nucleotide primers (5′-AAACTCGAGTCACACGAATTAAGCAAAGC-3′ and 5′-TTTTTCTCGTTGCGCCAT-3′). To generate mutant constructs, *tagln-r.b*ΔBS1-3 > *kaede* and *tagln-r.b*ΔBS1-8 > *kaede*, putative Fox binding sites (RYAAAYA; Chen et al., 2016) were mutagenized by the PCR-based method as previously described (Oonuma and Kusakabe, 2019). Oligonucleotide primers used for the mutagenesis of fox binding sites (BS1-8) were: 5′-GGTACGgcccaAAGCAGGAATTTTAATAGCAGT-3′ and 5′ -CTGCTTtgggcCGTACCTTTACCTTACTGGGTGG-3′ for BS1; 5′-GCTTCTcgggtTCTTGCCAAATAAGGCGA-3′ and 5′-GCAA GAacccgAGAAGCACGAAGCAAATTC-3′ for BS2; 5′-AACT GTTtgggtTCTTGGGCGAGCTAAGC-3′ and 5′-CCCAAGAac ccaAACAGTTTCATTGAAAGAGCC-3′ for BS3; 5′-GCAAA AGacccgATTCGTGCGACGGATTC-3′ and 5′-CACGAATcgggt CTTTTGCTCTCCCATGCA-3′ for BS4; 5′-CCTAGATcgggcTC GTACAACAGTTTGACGTAAGTTC-3′ and 5′-TGTACGAgc ccgATCTAGGCGTATTTCCACACG-3′ for BS5; 5′-TGTTATG gcccgACTCCATTCGTTCAACTTTCTAGA-3′ and 5′-GGAGT cgggcCATAACACCATACCTGTCGCGCG-3′ for BS6; 5′-GCGTTTtgggcCGTTTGATTGATAAATGTACGTAAGAGA-3′ and 5′-CAAACGgcccaAAACGCATTTAAAAGCCAGTT-3′ for BS7; 5′-CCTCATAGacccaAGCGAATCCATTGTCAAGTC-3′ and 5′-ATTCGCTtgggtCTATGAGGAGTATAGGCGAGGTG-3′ for BS8. To make the *pax2/5/8.a > kaede* plasmid, the 4.4-kb upstream region of *Ciona pax2/5/8.a* (Gene Model ID KH.S545.1/KH.S1363.2; Imai et al., 2004) was amplified from the genomic DNA of *C. intestinalis* type A by PCR using a pair of nucleotide primers (5′-CGACTCTAGAGGATCCGTGATTGTTACGTGG-3′ and 5′-TGGGGATCAGCAATGGATCCCCTTGCGGCC-3′), digested with *Bam*HI, and then inserted into the *Bam*HI site of the pSP-Kaede vector. Plasmid DNA constructs were electroporated into fertilized *Ciona* eggs as described by Corbo et al. (1997b).

### Immunofluorescence Staining

Immunofluorescent staining was carried out according to the method described by Nishitsuji et al. (2012). To visualize the localization of Kaede, a rabbit anti-Kaede polyclonal antibody (PM012; Medical & Biological Laboratories, Nagoya, Japan; for Kaede) was diluted 1:1000 in 10% goat serum in T-PBS (0.1% Triton X-100 in PBS) and used as the primary antibody. The secondary antibody was an Alexa Fluor 488-conjugated anti-rabbit IgG (A11008; Thermo Fisher Scientific, Waltham, MA, United States). Fluorescent images were obtained by using a laser scanning confocal microscope (FV1200 IX83; Olympus, Tokyo, Japan). Confocal images were collected at 1-μm intervals in the *z*-axis.

### Whole-Embryo Single-Cell Transcriptomic Analysis

A published single-cell transcriptome dataset of mid-tailbud embryos obtained using the 10x Genomics Chromium system (Horie T. et al., 2018; Horie R. et al., 2018; Cao et al., 2019) was used to analyze expression profiles of genes in the midline tissues. The dataset is available through GEO (GSE120035): https://www.ncbi.nlm.nih.gov/geo/query/acc.cgi?acc=GSE120035. The t-distributed stochastic neighbor embedding (t-SNE) analysis was performed using the Loupe Cell Browser 3.1.1 software (10x Genomics, Pleasanton, CA, United States). The processed data in a Loupe Cell Browser file (.cloupe) is available through the Mendeley data repository: http://dx.doi.org/10.17632/n4pxpr28cb.1. Differentially expressed genes were identified and ranked by statistical significance as previously described (Horie T. et al., 2018).

### Time-Lapse Live Imaging and Photo-Conversion of Kaede

Embryos electroporated with *pax2/5/8.a* > *kaede* were reared in ASW and mounted on a glass slide with ASW containing 1.5% methylcellulose at 10 hpf. Photoconversion of Kaede was performed as described (Oonuma et al., 2016). Fluorescent images were taken every 15 min for 12.5 h at 18°C by using a laser scanning confocal microscope (FV1200 IX83; Olympus, Japan). Confocal images were collected at 1-μm intervals in the *z*-axis.

## Data Availability Statement

The original contributions presented in the study are included in the article/Supplementary Materials, further inquiries can be directed to the corresponding author.

## Author Contributions

TK conceived the project and wrote the manuscript. KO, KS, TH, and TK designed the experiments. KO, MY, NM, NO, MM, ST, HS, EN, YH, and KS performed the experiments. TH, ST, MS, and TK analyzed the single-cell RNA-seq data. TH provided the essential materials. KO, NO, and TK analyzed and interpreted the data. KO, TH, and TK edited the manuscript. All authors reviewed the manuscript.

## Conflict of Interest

The authors declare that the research was conducted in the absence of any commercial or financial relationships that could be construed as a potential conflict of interest.

## Supplementary Material

Supplementary Video 1 Time-lapse of an embryo showing migration of mesenchyme cells labeled with photo-converted Kaede shown in Figure 5.
